# Post-Herpetic Neuralgia: Review of Pathophysiology, Mechanisms, and Drug Treatment

**DOI:** 10.2174/011570159X396405250924103619

**Published:** 2025-10-27

**Authors:** Xin Yan, Yufan He, Yuwan Yue, Chuan Zhang, Hanfeng Yang, Peilin Zhao

**Affiliations:** 1 Nanomedicine Innovation Research and Transformation Institute, Affiliated Hospital of Clinical School of Medicine, North Sichuan Medical College, Nanchong, Sichuan, 637000, China;; 2 Institute of Neurological Diseases, Affiliated Hospital of Clinical School of Medicine, North Sichuan Medical College, Nanchong, Sichuan, 637000, China;; 3 Biotechnology Innovation Drug Application and Transformation Key Laboratory of Sichuan Province, North Sichuan Medical College, Nanchong, Sichuan, 637000, China

**Keywords:** Herpes zoster, neuropathic pain, drug therapy, latent infection, pathogenesis, neural circuit

## Abstract

Post-herpetic neuralgia (PHN) is the most common complication of Herpes zoster infection. Although numerous targeted therapeutic drugs have been developed, it is difficult to achieve a complete cure. Abnormalities in neural circuits, ion channels, inflammatory factors, and gene regulation are crucial factors contributing to the development of PNH; however, the underlying mechanism remains unclear. Therefore, a comprehensive understanding of the underlying mechanisms of PNH is critical for advancing research and developing novel therapeutic strategies. Based on the latest findings, we systematically reviewed the current understanding of PHN mechanisms and corresponding treatment options and provided a comprehensive reference for future studies.

## 1 INTRODUCTION

As a common chronic neuropathic pain, post-herpetic neuralgia (PHN) is primarily triggered by varicella-zoster virus (VZV) infection. VZV remains dormant within the ganglia and is reactivated in particular situations, subsequently resulting in the onset of PHN. According to statistics from the Centers for Disease Control and Prevention (CDC) in the United States [1], approximately one million adults are diagnosed with herpes zoster (HZ) annually, with over half subsequently experiencing symptoms of PHN{Isagulyan, 2023 #67}. Studies in the Asia-Pacific region [2] suggest that the risk of HZ patients deteriorating to PHN ranges from 6.2% to 52.0%, and the ratio was 29.8% in a Chinese study [3]. Age is a crucial contributor to this phenomenon [4].

Additionally, prodromal pain, severe acute pain, significant skin lesions, and HZ ophthalmopathy (HZO) are critical for PHN [5]. Trigeminal HZ induces PHN and is limited to a single segment [6]. Compared to non-ophthalmic HZ, HZO patients with keratitis, conjunctivitis, or uveitis exhibit a 2-fold or higher [5] risk of progression to PNH [7, 8]. PNH mainly affects the ophthalmic division of the trigeminal nerve, whereas HZO affects the nasal nerve involved in the second or third branches of the trigeminal nerve (V2, V3) (approximately 37.5%) [9, 10] and does not increase the risk of PHN [11]. Ocular complications of HZ include inflammation, keratitis, uveitis, glaucoma, and acute and chronic retinal necrosis [12]. Dermatomes and ganglia, particularly around the eyes, are most severely affected by HZ and easily develop into PHN. In a cohort study [13], 28% of the patients with HZO experienced severe rashes, and a heightened risk of PHN may play a critical role. There is currently no consensus regarding the pathogenesis of PHN. However, various hypotheses have been proposed by various studies, including central and peripheral sensitisation, ion channel alterations, and metabolic factors. Recent studies have identified gene-level changes in patients [14]. In addition to persistent neuralgia, clinical manifestations of PHN include rashes and chronic pruritus [15]. The use of traditional therapies, including tricyclic antidepressants, pregabalin, and antiviral drugs, remains prevalent. Interventional therapy has emerged as a pivotal approach in the management of refractory cases. Research focused on hub proteins, including human microplasminogen (Plg), apolipoprotein A1 (ApoA1), and apolipoprotein E (ApoE) as well as their related cholesterol metabolism and coagulation processes within the cerebrospinal fluid of patients has revealed potential therapeutic pathways [16]. Moreover, botulinum neurotoxins (BoNTs), a family of neurotoxins produced by *Clostridium* and related bacteria, have gained recognition as safe and effective treatments for chronic neuropathic pain, including PHN that specifically targets nociceptive pathways. Botulinum toxin A (BTX) is a novel therapeutic agent for post-herpetic pruritus (PHI) [17] (Table **1**). Pain is a multi-dimensional experience encompassing motivational, emotional, cognitive, evaluative, and sensory-discriminative dimensions. This article discusses the sensory-discriminative mechanisms and pathways underlying pain. It integrates potential mechanisms and hypotheses regarding the development of PHN and provides a comprehensive summary of various treatment strategies and preventive measures. We also explored both conventional and non-pharmacological approaches that are currently in use. Furthermore, we identified potential drug targets, underscoring the importance of enhancing diagnostic and therapeutic strategies to address unmet clinical needs within this domain.

This review employed a rigorous methodological framework to investigate the core mechanisms of post-herpetic neuralgia (PHN) and its therapeutic interventions. The search strategy incorporated both controlled vocabulary (*e.g*., “post-herpetic neuralgia”, “pathophysiology”, “drug treatment”, “herpes zoster”) and free-text terms across multiple authoritative databases, including PubMed, Web of Science, and Cochrane Library. Furthermore, manual screening of high-impact journals (*e.g*., *Pain*, *Brain*, *Nature Medicine*) was conducted. The review encompassed literature published from 1988 to 2024, with particular emphasis on high-quality studies from the most recent decade (2014-2024). The scope included fundamental research (animal models, molecular mechanisms), clinical investigations (randomized controlled trials, cohort studies), meta-analyses, and guideline consensuses. The literature selection process followed a multi-stage approach: initial screening based on titles and abstracts by team members to exclude clearly irrelevant studies, followed by full-text review of potentially eligible articles. During data extraction, priority was given to multicenter randomized controlled trials (*e.g*., comparative studies of magnesium versus ketamine analgesia), large-scale cohort studies (*e.g*., epidemiological data from the US CDC), and high-quality meta-analyses (*e.g*., Cochrane reviews of antiviral therapies). To ensure scientific rigor and reliability, case reports, non-peer-reviewed literature, and studies with conflicting findings (*e.g*., corticosteroid efficacy) were excluded, with conclusions drawn only after cross-validation of multiple studies.

## 2 POTENTIAL MECHANISMS AND CORRESPONDING TREATMENTS OF PHN

### 2.1 PHN and Symptom Characteristics

HZ is attributed to the local reactivation of the varicella-zoster virus (VZV), leading to a unilateral painful rash in the skin of patients with chickenpox or varicella [18]. The virus remains latent within the dorsal root ganglia (DRG) of the spine or sensory ganglia of the cranial nerves for a long period of time. As immune function declines with age, the virus may reactivate and transport axons to skin cells, resulting in HZ. The incidence of HZ has increased since the 2000s; however, there is no national or international consensus on the cause of this increase [19-21]. Additionally, certain investigators observed HZ reactivation after COVID-19 vaccination [22]. Neuralgia is the most common clinical manifestation of PHN. Patients describe three types of unbearable pain that include (1) spontaneous, persistent, throbbing, and burning pain, (2) stabbing, shooting, shock-like, and paroxysmal pain, and (3) severe pain caused by non-nociceptive stimuli (hyperalgesia) [23]. HZ pain tends to resolve spontaneously over time [24], but this can take months to years [25]. Therefore, adequate pain treatment is essential for improving the patient's quality of life. Furthermore, the diagnosis of HZ in some patients may be quite challenging, given the diversity of clinical presentations and the atypicality of cases [26].

#### 2.1.1 Pathological Features

Acute herpes zoster triggers cutaneous inflammation accompanied by partial nerve disuse and an inflammatory reaction with haemorrhagic necrosis of the DRG. During the course of the disease, the central nervous system also exhibits significant pathological changes, and autopsy reports indicate that patients with PHN commonly present with atrophy of the spinal dorsal horn [24]. Histological examination further revealed that the peripheral and central nervous systems of PHN patients suffered myelin sheath and axon loss and atrophy of the dorsal horn [27]. Watson *et al*. [28] compared epidermal axonal density between patients with PHN and those with self-limited HZ. They observed that the epidermal axonal density was significantly reduced in most patients.

### 2.2 Herpes Virus Latency and Reactivation and Related Treatments

#### 2.2.1 Mechanism of Herpes Virus Reactivation

**Fig. (1):**
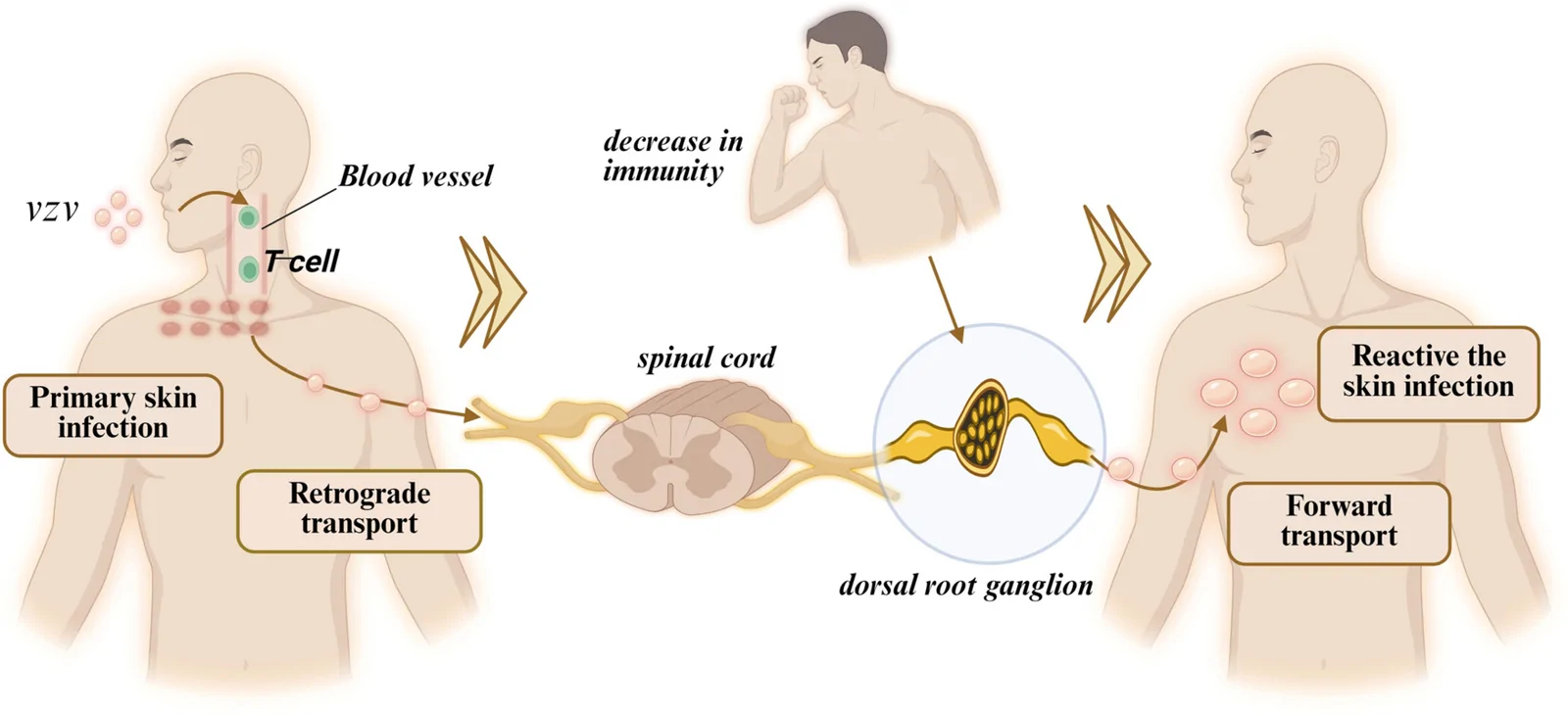
The pathogenesis and progression of HZ.

The physiological process of PHN has been elucidated in detail by scientists and clinicians over the past decades, and reactivation of the herpes zoster virus is a direct cause of PHN formation. Studies have demonstrated that patients are usually infected with the varicella virus during childhood. The virus is not completely eliminated after healing, and it lurks in the sensory ganglia of the body and reactivates to cause viral diseases when the body's immunity is lowered [29, 30]. In general, the immune system can prevent the re-emergence of VZV by generating memory cells and antibodies against the virus. However, as individuals age or experience immunosuppression, cellular immunity diminishes, permitting the clinical reactivation of the virus. Consequently, the virus reactivates nerve-related satellite cells and spreads to the skin through the peripheral nerves [24], triggering HZ symptoms (Fig. **1**).

The left side of the figure illustrates the early stages of varicella infection, in which the virus penetrates the body *via* the skin and establishes latency within the dorsal root ganglia (DRG). Subsequently, the virus is reactivated and migrates *via* nerve fibers to the anterior root ganglion of the spinal cord. The stage at which HZ emerges is depicted on the right, where the virus reawakens and travels anterogradely along the nerve axons to the skin, resulting in the characteristic symptoms of HZ.

#### 2.2.2 Drug Treatments

The optimal approach for managing PHN involves suppression or eradication of the underlying virus. Antiviral drugs, such as acyclovir (Table **1**), famciclovir, and vasiclovir, which are nucleoside analogues that inhibit the replication of human herpes viruses (including VZV), are widely used in clinical practice. When taken orally, these drugs reduce the duration of viral shedding, accelerate rash healing, reduce the severity and duration of acute pain [31, 32], and decrease the likelihood of developing PHN. Given its safety profile and favourable tolerance, antiviral therapy should be considered in all individuals with HZ [33]. Additionally, corticosteroids can be used in conjunction with antiviral medications to further mitigate the severity and duration of the illness [34, 35]. Nevertheless, considering the multiple adverse effects of corticosteroids, they should be used only in patients presenting with severe symptoms or without major contraindications.

#### 2.2.3 Current Status of Vaccine Prevention

Vaccine prophylaxis is the most effective method of protection against PHN [22]. A systematic review has indicated that the live HZ vaccine exhibits optimal protective efficacy in the early stages and, despite a significant decrease in efficacy over time, retains some protective capacity even ten years after vaccination. Over the past few decades, the protective efficacy of the vaccine against HZ has decreased, but its efficacy against PHN has increased [36]. Currently, there are two types of PHN vaccines, including live attenuated vaccines and a new adjuvant recombinant VZV vaccine [37]. Both vaccines have demonstrated safety and efficacy in clinical trials involving immunocompetent healthy adults, immunocompromised patients, and patients with immune disorders. Compared to the live attenuated HZV vaccine, the recombinant HZV vaccine demonstrated greater effectiveness in preventing HZ. Additionally, recombinant HZV vaccines do not possess replicative capabilities, making them safe for use in immunocompromised individuals [38, 39]. However, the efficacy of these two vaccines in preventing PHN has not met expectations and long-term market surveillance data [13]. Extremely rare adverse events following recombinant HZV vaccination include optic neuritis associated with myelin oligodendrocyte glycoprotein [40].

### 2.3 Abnormalities of the Neural Circuits

As a common chronic pain, PHN involves multiple regions of the central and peripheral nervous systems during its formation and occurrence, leading to damage or abnormal activity in related nerve circuits.

#### 2.3.1 Peripheral Nervous System Injury and Sensitization and Related Treatment

##### 2.3.1.1 Damage to the Peripheral Nervous System

According to the physiological mechanism of PHN, the peripheral nervous system is the first to suffer from these effects, including abnormal discharge of damaged peripheral fibers, confusion of nerve excitation transmission, and abnormal excitation of sympathetic nerves. Studies have demonstrated that approximately 60% of patients suffer impaired nerve conduction and sensory loss [41]. In some patients, there is an increased response to abnormal skin stimuli along with a characteristic loss of light sensation [42]. In cases of allodynia, functional impairment of skin C fibers is inversely proportional to the intensity of neuralgia, suggesting that allodynia signals are not directly associated with C fiber sensitisation. Notably, other patients who lose large and small fibers experience spontaneous severe pain and have no anomalous pain or nociceptive sensitisation caused by the afferent impairment of central neurons [43].

##### 2.3.1.2 The Core Function of the DRG

The DRG is located between the intervertebral foramina of the spinal cord, and its sensory neurons are key nodes in the pain-afferent pathway in the cerebral cortex [44]. This is crucial for the latency of the HZ virus and is considered a core factor in the induction and maintenance of PHN. According to the “ectopic pacing hypothesis”, the main mechanism of pain after nerve injury is the generation of ectopic pulses at the injury site or in the DRG. Studies conducted on both animals and humans have observed that central sensitisation leads to sensory recognition of systemic skin Aβ afferent fibers. Additionally, studies have revealed that blocking the DRG reduces spontaneous pain, whereas blocking the distal DRG is ineffective, suggesting that the primary pain source in PHN may be the DRG [23, 45].

##### 2.3.1.3 Pathological Model Classification

PHN can be divided into irritable nociceptors and deafferent models. During viral reactivation, the virus replicates from the DRG and spreads peripherally, triggering immune responses and inflammation-damaging peripheral nerves, ultimately reducing the threshold of neuronal inhibition of pain and pain signal depolarisation and leading to pain sensation triggered by non-painful stimuli, such as peripheral sensitisation. Studies have also determined that an increased number and alteration of voltage-gated ion channels in damaged peripheral nerves in HZ rats increased the peripheral hyperexcitability of injury receptors in patients with PHN [46]. During HZ, dormant viruses reactivate and propagate in immunocompromised states, such as fatigue and influenza, reaching dermatomes along the infected nerve and causing rash formation [47]. Both central and peripheral neurons are damaged during this process. As a result, damaged peripheral neurons lose the ability to inhibit nociceptive pain signals due to the immune response and inflammation, and this easily triggers nociceptive pain and generates ectopic discharges, leading to nociceptive hypersensitivity and ectopic pain [41, 48].

##### 2.3.1.4 Traditional Drug and Physical Therapies

The peripheral nervous system is a key target for the treatment of PHN. Drug therapy is the preferred treatment for PHN. For example, doxorubicin, an antitumour drug, can cause necrosis and damage signalling pathways when injected into the intervertebral foramen at low concentrations and doses. Disruption of the DRG with adriamycin proved to be an effective and safe intervention in a 72-patient controlled study without any observed adverse reactions [49, 50]. Additionally, computed tomography (CT) -guided radiofrequency ablation of the cervical DRG can reduce the visual analogue scale (VAS) pain score and skin sensation and significantly reduce allodynia [51]. Interventional surgery has become the basis for treating refractory diseases, and experienced experts have achieved remarkable results using a multimodal approach [52]. Transcutaneous electrical nerve stimulation is a safe and non-invasive method of pain control that inhibits dorsal horn segments and descending pathways while stimulating endogenous opioid secretion [50]. Two randomised controlled trials evaluated the efficacy of Transcutaneous Electrical Nerve Stimulation (TENS) versus PHN drugs using high-frequency TENS for 30 min daily for 1-2 months. TENS with a subcutaneous injection of pregabalin was effective in the fourth week, whereas TENS with topical cobalamin demonstrated effective analgesia [49]. However, the efficacy of TENS alone is unclear. Interestingly, TENS may be critical for preventing acute herpetic neuralgia, particularly during the first to 3-6 months of treatment [53]. The analgesic mechanism of TENS is based on the gating theory. When afferent fibers are stimulated, the input of small pain afferent fibers to dorsal horn neurons is inhibited before projection to the spinal cord. The effect of a temporary nerve block with a local anaesthetic injection on the control of neuropathic pain is controversial [54]. Neurosurgery is the last resort for the treatment of intractable pain. Studies have demonstrated that electrical stimulation of the thalamus and anterolateral myelotomy can alleviate symptoms in patients with PHN [33]. Spinal cord stimulation is an established treatment option for patients with pharmacologically resistant PHN. High-frequency burst and DRG stimulations are promising methods for treating PHN [55]. High-voltage pulsed radiofrequency surgery (PRF) is superior to standard-voltage PRF for improving the analgesic effect in patients with PHN and does not increase the incidence of treatment-related adverse effects [56].

##### 2.3.1.5 Neurotoxins and New Therapies

BoNTs, a family of neurotoxins produced by bacteria, such as *Clostridium difficile*, have been approved for use in a variety of treatments, including the treatment of neuropathic pain, such as PHN and trigeminal neuralgia. Peripherally administered BoNTs are absorbed by nerve terminals and reduce peripheral neurogenic inflammation by decreasing the release of glutamate, calcitonin gene-related peptides, and substance P. BoNTs reduce the expression of sensory genes in the sensory ganglia, and the release of neurotransmitters and neuropeptides from the central terminals of primary afferents that may lead to reduced central sensitisation of the spinal dorsal horn or trigeminal nucleus. To enhance their therapeutic potential, BonTs that specifically target the injury perception pathway have been developed to treat chronic pain. Further mechanistic studies on BoNT-induced analgesia could improve the use of BoNTs for the treatment of neuropathic pain and enhance their safety and efficacy [17]. BTX, a neuromodulator that inactivates SNARE proteins and inhibits acetylcholine release from presynaptic vesicles, is a new treatment modality for PHI [57]. Additionally, one study observed that BoNT/A could attenuate DRG neuronal focal death in rats by upregulating cyclic AMP inhibition by ELANE, thus demonstrating its efficacy for PHN treatment [57].

#### 2.3.2 Central Sensitization

**Fig. (2):**
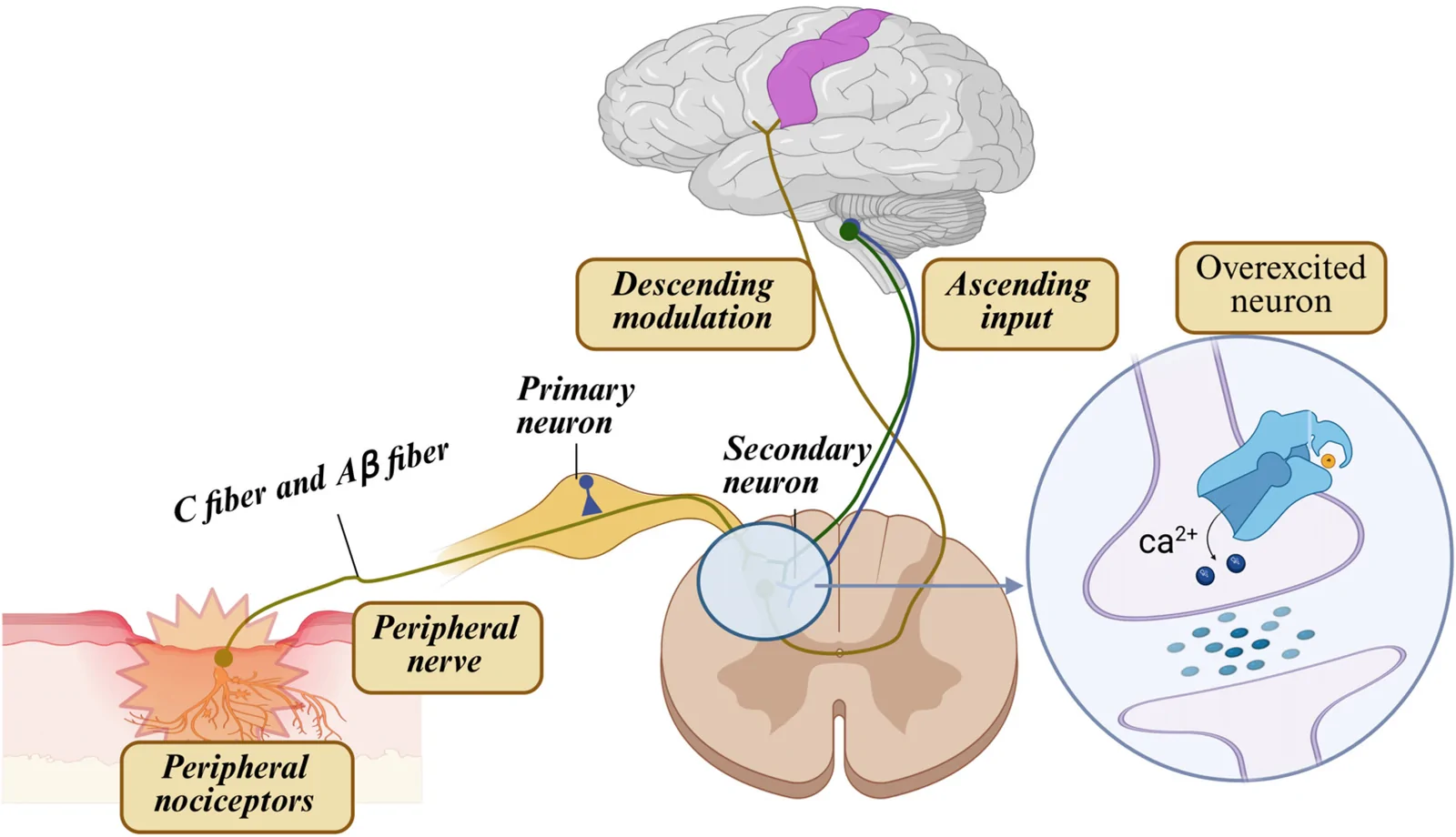
Schematic diagram of central sensitization of chronic pain.

While peripheral sensitisation is a key driver of PHN, central sensitisation further amplifies pain signals through the spinal and supraspinal pathways. Varicella-zoster virus infection induces neuroinflammation that damages the spinal dorsal horn and pain-inhibitory pathways to relieve pain. This process may subsequently result in central nervous system (CNS) sensitisation *via* tight interconnections [58, 59]. The progression and persistence of CNS sensitisation depend upon the release of glutamate (a ligand of the N-methyl-D-aspartate receptor NMDA) and substance P from sensitised C-fibers, and this leads to increased pain sensitivity of secondary neurons in the dorsal horn [41]. Most studies have demonstrated that dynamic mechanical allodynia (DMA) is a central sensitisation mediated by low-threshold Aβ fiber afferents and that complete innervation of large fiber nerves is crucial in neuropathic pain. In the context of central sensitisation, the mechanisms by which large fiber inputs are integrated into the nociceptive transduction pathways remain unclear. Possible mechanisms include sprouting of Aβ fibers to spinal cord layer II, phenotypic switching, disinhibition of established pathways, loss of inhibition of low-threshold mechanosensory C-tactile afferents, disruption of chloride ion mediated spinal inhibition, and an imbalance between the altered firing frequency of Aβ fibers and nociceptive inputs (Fig. **2**) [60]. The neuroinflammation induced by VZV infection would relieve pain but damage the spinal dorsal horn and inhibitory pathways, resulting in central nervous system sensitization.

The medical applications of magnesium can be traced back to the 17^th^ century. Owing to its ability to inhibit calcium ion influx by antagonising NMDA receptors, many studies have explored its potential value as an auxiliary analgesic drug [61]. Magnesium sulphate is as effective as ketamine in controlling pain associated with chronic PHN [62]. Magnesium sulphate is regarded as an emerging potential treatment option for pain management in PHN. However, further direct evidence is required to establish magnesium sulphate as an optimal treatment for PHN [63].

### 2.4 Changes in Ion Channels

#### 2.4.1 Sodium Channels

The transmission of pain signals depends upon precise communication within and between neurons, and changes in ion channels play a crucial role in the generation and transmission of pain. Studies have demonstrated that the voltage-gated sodium channels Nav1.1, Nav1.6, Nav1.7, Nav1.8, and Nav1.9 are distributed in different patterns in peripheral sensory neurons and are key factors in the regulation of sensory nerve excitability [64, 65]. For example, Nav1.8 accumulates at the nodes of Ranvier and is overexpressed in neuromas [60], and it is a highly sensitive spherical structure at the ends of severed nerve fibers. Recently, significant progress has been made in linking mutations in voltage-gated sodium channels to various pain disorders. Upregulation and increased density of membrane sodium channels have been observed in the proximal part of injured DRG axons. Six sodium channel isoforms are present in DRG neurons, of which isoforms, such as NaV1.7 and NaV1.8 (specific isoforms), are specific to sensory neurons and are not distributed in other regions of the nervous system. These channels are difficult to detect in normal DRG neurons and recover rapidly after inactivation, promoting low-threshold repetitive firing of injured neurons. This is thought to be the mechanism underlying ectopic impulse generation. Therefore, blocking these sodium channel subtypes is an important strategy for treating neuropathic pain [54]. Tricyclic antidepressants (TCAs) exert significant therapeutic effects by inhibiting the reuptake of serotonin and norepinephrine while acting as antagonists of voltage-gated sodium channels and α-adrenergic receptors to regulate the corresponding descending pathways [23]. Lidocaine, as an amide local anaesthetic, mainly acts on voltage-gated sodium channels on unmyelinated C-fibers and myelinated Aδ-fibers, and it stabilises neuronal membrane potential, reduces ectopic discharges, and is capable of temporarily blocking the conduction of nerve fibers, exerting an anaesthetic effect and elevating the pain threshold and relieving local pain. Clinically, lidocaine has been widely used due to its rapid onset, strong penetration and diffusion abilities, long duration of action with less irritation, and low toxicity [7].

#### 2.4.2 Calcium Channels

**Fig. (3):**
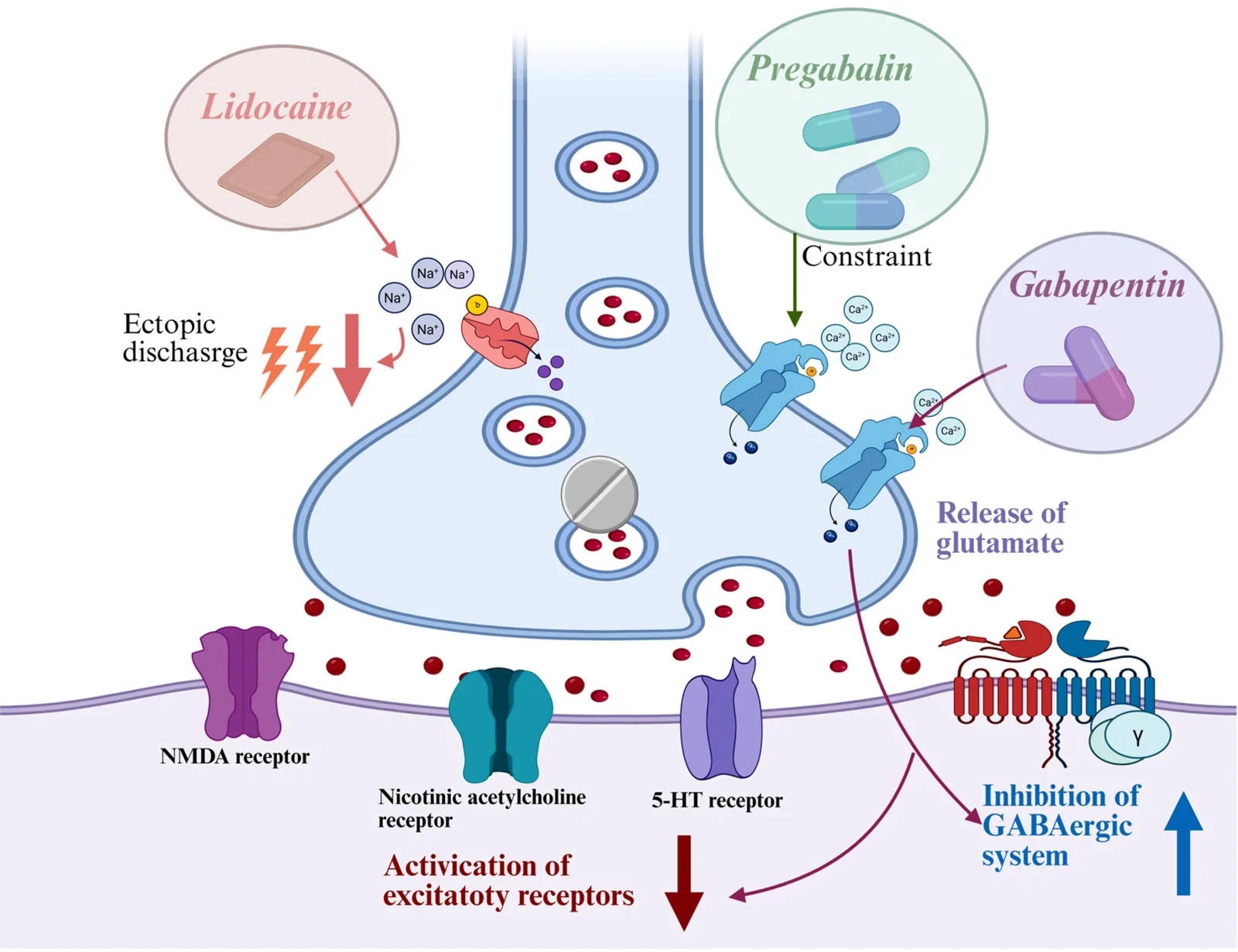
Pharmacological targets of PHN drugs, including sodium/calcium channel blockers and TRPV1 agonists.

In addition to sodium channels, calcium channels play an indispensable role in pain conduction. Calcium enters nerve endings through calcium channels and regulates the activity of related growth proteins. Recent *in vitro* studies have revealed that N- and L-type calcium channels play important roles in the release of calcitonin gene-related peptide (CGRP) from injured nerve endings. It was further indicated that inhibition of N-type, T-type, and P-type calcium channels can effectively block the development of experimental neuropathic pain [54]. Among the many calcium channel subtypes, the Cav3.2 isoform has become a research hotspot due to its expression in the DRG and spinal cord neurons. In Spared nerve injury (SNI) and other traumatic nerve injury models and chemotherapy-induced neuropathy models, the expression level of Cav3.2 is significantly increased, along with the current amplitude and density of T-type calcium channels [60]. Calcium channel regulation is an important target for the clinical treatment of PHN. Pregabalin and gabapentin, which are gamma-aminobutyric acid (GABA) derivatives, are the only first-line oral drugs approved by the US Food and Drug Administration (FDA) for treating PHN [66, 67]. These drugs inhibit the activation of pain-signalling neurons by binding to voltage-gated calcium channels in the presynaptic horn, reducing the release of excitatory neurotransmitters such as glutamate and substance P [68]. Clinical trials have demonstrated that pregabalin may be more advantageous than gabapentin for treating patients with PHN [69], significantly reducing pain levels and sleep disturbances. However, pregabalin may also cause systemic and central nervous system (CNS) toxic effects, such as somnolence, dizziness, dry mouth, and blurred vision (Fig. **3**). Crisugabalin (HSK16149), a novel ligand for the voltage-gated calcium channel α2δ-1, is a highly selective oral γ-aminobutyric acid analogue. Preclinical studies have demonstrated that HSK16149 exhibits significant analgesic effects, long-term efficacy, and minimal central adverse effects. Similar to pregabalin, kligabalin inhibits Ca^2+^ influx in the CNS by binding to α2δ-1 and reduces the release of excitatory neurotransmitters. Its binding affinity to α2δ-1 is greater than that of pregabalin [70], and its brain tissue exposure level is only 1/18 of that of pregabalin, indicating milder neurotoxicity [71]. A randomised clinical trial conducted in a Chinese population demonstrated that crisugabalin was well tolerated by healthy participants [72] and has the potential to be the treatment of choice for chronic neuropathic pain. The capsaicin receptor (TRPV1) is a novel ion channel that is widely distributed in the cell membranes of mammalian sensory neurons. Its activation mechanism involves the induction of cation influx (such as Ca^2+^) upon receptor activation, and this leads to neuronal excitation. In the treatment of PHN, approved capsaicin exerts its effects by binding to cutaneous nociceptors, particularly the TRPV1 receptor, to regulate the transcellular movement of sodium and calcium ions. This process modulates the opening of ion channels, thereby influencing neural signal transduction and achieving analgesic effects. This mechanism not only highlights the critical role of TRPV1 in sensory transmission but also elucidates the scientific rationale for capsaicin-based therapy targeting ion channels in neuropathic pain management [23]. Furthermore, researchers determined that capsaicin pretreatment improved survival and suppressed inflammatory responses in VSV-infected mice while reducing VSV replication in the liver, lung, and spleen. This suggests that capsaicin is a potential small-molecule drug candidate, providing a feasible pharmacological strategy to enhance resistance against viral infections [73]. Repeated exposure to capsaicin can deplete substance P and neuropeptides, and this reduces the pain response and ultimately achieves analgesia [74]. Researchers have suggested that TRPV1 receptors are promising targets for analgesic drug development (Table **1**) [75].

The figure illustrates the action mechanisms of drugs, such as lidocaine, pregabalin, and gabapentin. Lidocaine acts on “ectopic discharge”, while pregabalin affects glutamate release through “constraint” and inhibits the GABAergic system [66, 69]. Additionally, it involves the activation of excitatory receptors, such as the NMDA receptor, nicotinic acetylcholine receptor, and 5-HT receptor, demonstrating the drugs’ intervention process in nerve signal transmission-related links.

### 2.5 Immune Cells and Inflammation

Studies have revealed that immune cells and their derived factors, such as tumour necrosis factor-α, various interleukins (including IL-10, IL-1β, IL-4, and IL-17), and interferon-γ, play important roles in pain. T cells play a decisive role in the development of neuropathic pain. Studies have demonstrated that mice lacking T-cells are unable to develop neuropathic mechanical allodynia following nerve injury [76]. Genomic screening revealed that the serine protease inhibitor Serpina3n is a key molecule determining resistance to neuropathic pain in rats and mice, and it was determined that Serpina3n exerts its effects by inhibiting the pro-injurious action of T-cell-derived leukocyte elastase in peripheral sensory neurons [77]. Genetic deletion and pharmacological inhibition of leukocyte elastase have exhibited inhibitory effects on allodynia and spontaneous pain in various forms of neuropathic pain, including nerve injury, diabetic neuropathy [77], cancer pain involving nerve remodelling, and osteoarticular inflammatory pain [30]. Additionally, as a pro-inflammatory cytokine, IL-18 plays a crucial regulatory role in the immune response and inflammatory processes. It also plays an important role in the regulation of glial cell activity in the DRG. Liang *et al*. evaluated the effect of IL-18 protein levels on the occurrence of PHN using bidirectional MR analysis and observed a causal relationship between increased IL-18 protein levels and the occurrence of PHN [78]. These findings provide new perspectives for identifying and protecting at-risk individuals for PHN and offer the possibility of developing new approaches for PHN prevention and treatment. Macrophages also play an important role in the pathophysiology of neuropathic pain [79-81]. Recent studies have observed a strong clinical association between macrophage activation and peripheral analgesic effects of angiotensin receptor 2 antagonists, particularly in the absence of receptor expression in sensory neurons. Angiotensin receptor 2 expression on macrophages invading the site of nerve injury can mediate the attenuation of abnormal neuropathic pain [60, 82]. Targeting immune cells and their mediators exhibits great potential for the treatment of neuropathic pain, particularly with respect to its peripheral components.

### 2.6 Metabolites, Hypoxia, and Mitochondrial Factors

A common element of the human condition and animal models of different types of neuropathic pain is pronounced mitochondrial dysfunction in peripheral sensory neurons induced by direct nerve injury or mitochondrial toxicity induced by chemotherapy, anti-HIV treatment, and high glucose and its metabolites in diabetic neuropathy [83]. Mitochondrial dysfunction and the production of reactive oxygen species (ROS) are closely linked and lead to energy deficits and degeneration. Peripheral sensory neurons with long axons and high-energy demands are particularly vulnerable to injury-induced bioenergetic crises. Recent studies have also demonstrated that mitochondrial toxicity and the deleterious effects of ROS are not limited to peripheral nerves in neuropathic pain. Following peripheral nerve injury, mitochondrial superoxide levels increase in the spinal dorsal horn through a superoxide dismutase-2-dependent mechanism, leading to an increase in the frequency of miniature excitatory postsynaptic currents in spinal excitatory neurons [79]. Thus, overexpression of superoxide dismutase-2 leads to nerve injury-induced neuropathic allodynia. The mechanisms regulating mitochondrial toxicity and reactive oxygen species (ROS) production are only beginning to be analyzed in the context of neuropathic pain. PINK1 (PTEN-induced putative kinase 1) is a protein kinase localized in the mitochondria, and studies have revealed its potential neuroprotective role. Dysfunction of PINK1 impairs the removal of damaged mitochondria, leading to mitochondrial dysfunction, oxidative stress, and neuronal degeneration [84].

Guo *et al*. [85] screened 2,801 compounds from over 200 Asian plants through a literature review. Virtual screening grouped the compounds by binding energy, and this was followed by further screening *via* MM/PBSA that identified vitexin, luteoloside, and 2'-deoxyadenosine-5'-monophosphate with high scores of -59.439, -52.421, and -47.544 kcal/mol, respectively. Mechanistic validation using pain behaviour quantification, ELISA, qPCR, western blotting, and transmission electron microscopy demonstrated that vitexin exerted the most significant therapeutic effect on PHN rats (followed by luteoloside), while 2'-deoxyadenosine-5'-monophosphate exerted no significant effect. This study confirms that vitexin relieves PHN by regulating PINK1-mediated mitophagy. However, this study is in its early stages, and further research is necessary to understand the mechanisms of action, individual differences in response, safety, and comparative efficacy with established treatment regimens.

### 2.7 Gene Regulatory Relevance

Extensive HSV-1 directly triggers neuroinflammation, nerve damage [86], and the whole-cell death of microglia, leading to massive lysis of microglia and further neuroinflammation [87] that induces PHN. Recently, it has been demonstrated that HSV-1 infection significantly upregulates the expression of protein arginine methyltransferase 6 (Prmt6) in microglia in the dorsal horn of the spinal cord. Prmt6 inhibits the phosphorylation, dimerisation, and nuclear translocation of TBK1/IRF3 through methylation and inactivation of the stimulator of interferon gene (STING), ultimately reducing type I interferon (IFN-I) production [88]. The reduction in IFN-I impairs the ability of the body to inhibit and clear HSV-1, leading to an increase in the viral load, directly triggering neuroinflammation and nerve damage and promoting the development of PHN [89]. These findings suggest potential clinical strategies and therapeutic targets for treating PHN, providing a scientific basis for the development of new drugs targeting Prmt6. During PHN, cerebrospinal fluid (CSF) can activate glial cells and cause inflammation; however, the specific changes in CSF composition are not clear. Recent studies have revealed that central bone morphogenetic protein 4 (BMP4) levels increase during neuropathic pain (NP). Compared to normal CSF, the concentration of BMP4 was significantly increased in PHN-CSF, and this exacerbated abnormal pain and triggered the activation of microglia and astrocytes.

Additionally, PHN-CSF may induce microglial proliferation and pro-inflammatory transition, enhance iron storage, and activate astrocytes by activating the SMAD159 and STAT3 signalling pathways. These effects can be alleviated by the application of noggin, a secreted homodimeric glycoprotein [90]. Both Noggin and the STAT3 inhibitor Stattic significantly reduced BMP4-induced upregulation of glial fibrillary acidic protein (GFAP) and IL-6, as well as the phosphorylation of SMAD1/5/9 and STAT3 in cultured astrocytes. Additionally, microglia depletion reduced PHN-CSF-induced astrocyte proliferation, inflammation, and endogenous BMP4 expression.

Studies have also revealed that long non-coding RNA (lncRNAs) can bind to pSTAT3 and exert their regulatory effects by affecting the stability of the pSTAT3 protein [91]. KCNA2-AS binds to pSTAT3, and a reduction in KCNA2-AS can inhibit the transfer of pSTAT3 to the nucleus in activated rat spinal cord astrocytes [92], indicating that KCNA2-AS may regulate the activation of spinal astrocytes by regulating the cytoplasmic-to-nuclear transfer of pSTAT3, thereby affecting PHN. PHN may be associated with an abnormal response to post-herpetic skin injuries. The microRNA (miRNA) miR-16-5p plays an important role in multiple biological processes. Recent studies have revealed decreased expression of miR-16-5p, miR-20a-5p, miR-505-5p, miR-3664-3p, miR-4714-3p, and let-7a-5p in the skin of patients with PHN. In a resiniferatoxin (RTX)-induced PHN mouse model, downregulation of both miR-16-5p and let-7a-5p was observed in RTX-treated plantar skin, and this was consistent with the situation in PHN patients. These findings suggest that plantar miR-16-5p alleviates RTX-induced PHN-stimulated pain by inhibiting Akt3 expression in the skin [14]. Electroacupuncture (EA), a therapeutic modality that electrically stimulates specific acupoints in the human body, has been observed to reduce apoptosis and inflammatory responses in neuronal cells of PHN rats, serving as an excellent analgesic for the treatment of neuropathic pain [93]. It has been discovered that EA can not only mitigate the pathological changes in unmyelinated and myelinated fibers [94] but also inhibits autophagy in neuronal cells by increasing the expression of miR-223-3p, a mechanism that may be related to the targeting and regulating effect of miR-223-3p on Rab1 [95]. Human trials have demonstrated that EA effectively relieves pain and anxiety [96] with no observed adverse reactions [97]. Clinical studies combining gabapentin or pregabalin with EA are currently in progress [93, 98].

### 2.8 Effect of Gut Microbiota on PHN

In recent years, the intricate interactions between the gut microbiota (GM) and neurological disorders have received widespread attention [99], particularly their important role in the occurrence and regulation of neuropathic pain (NP) [100, 101]. Studies have demonstrated that there is a strong association between some specific components of the GM and NP and that intestinal bacteria are involved in the regulation of nociception through a variety of pathways, such as regulation of the immune system, modulation of inflammatory responses, and activation of neural pathways [102]. The GM can produce and release bioactive metabolites that may act as neuromodulators, interacting with the central nervous system and thereby affecting its operation. A recent study conducted a comparative analysis of 27 patients with PHN and 27 healthy individuals and revealed a preliminary association between the GM and the two groups [103]. By analysing the GM in stool samples, researchers observed significant differences in 37 bacterial genera between the two groups. These differences may be closely related to the clinical manifestations of PHN, suggesting that an imbalance in the gut microbiota may be a key factor in the pathogenesis of PHN. The complexity of the bidirectional communication pattern between the gut microbiota and the nervous system, mediated through multiple signaling pathways, highlights the importance of the gut-brain axis. The intricate microbial ecosystem in the gastrointestinal tract not only profoundly affects neural function but also allows feedback from the state of the nervous system on gut microbial composition and function. This interrelationship involves both the central and peripheral nervous systems, as presented in Fig. (**4**).

The gut-brain axis is the link between the brain and the gut, and it is also a neural structure independent of the central nervous system that connects and affects intestinal function through nerves. The gut microbiota affects the function of the gut-brain axis through immunomodulatory, vagus nerve regulatory, neuroendocrine regulatory, metabolite, and endocrine regulatory pathways. Given the crucial role of the GM in the regulation of neuropathic pain, its modulation has become an important strategy for the treatment of this condition. These strategies include but are not limited to using probiotics, faecal microbiota transplantation (FMT), dietary modifications, and vitamin supplementation. Providing emotional support to help patients effectively cope with the stress and emotional challenges associated with neuropathic pain is also important. These strategies not only optimise the balance of the gut microbiota but also positively impact nervous system health, thereby reducing the symptoms of neuropathic pain.

## 3 CONCLUSION AND PROSPECTS

### 3.1 Summary

Post-herpetic neuralgia is a common chronic neuropathic pain caused by VZV infection and is driven by viral latency reactivation, neural circuit abnormalities, ion channel alterations, immune-inflammatory responses, and gene-metabolic dysregulation. Clinically, it is characterised by persistent burning pain, electric shock-like sensations, and hyperalgesia lasting ≥3 months after herpes zoster rash resolution. Current therapies primarily involve antiviral agents and anticonvulsants complemented by interventional procedures and vaccine prophylaxis. However, personalised precision medicine strategies remain to be explored. The varicella-zoster virus latently resides in the dorsal root ganglia (DRG), reactivates during immunocompromise, and spreads along axons, causing organic injuries, such as DRG haemorrhagic necrosis, spinal dorsal horn atrophy, and nerve demyelination. Concurrently, the aberrant expression of sodium channels (Nav1.8/1.7) in DRG neurons triggers ectopic discharges in peripheral nerves, whereas C-fiber damage induces hyperalgesia. In the spinal dorsal horn, increased release of glutamate and substance P enhances neuronal excitability, and this is accompanied by abnormal sprouting of Aβ fibers to form a central sensitisation cascade. Additionally, proinflammatory factors, such as leukocyte elastase, secreted by T cells, and IL-18, exacerbate neuroinflammation. Meanwhile, mitochondrial dysfunction (*e.g*., PINK1-mediated autophagic abnormalities) and gut microbiota dysbiosis (with differences in 37 bacterial genera) contribute to disease progression through multisystem interactions. In terms of expanded treatment approaches, a stratified drug strategy is integrated with interventional therapies and novel preventive directions. In the acute phase, antiviral agents, such as acyclovir, are used to inhibit viral replication and reduce the risk of PHN, while in the chronic phase, pregabalin/gabapentin target the α2δ subunit of calcium channels, lidocaine patches block sodium channels, and capsaicin depletes substance P to alleviate pain. For refractory cases, botulinum toxin A (BTX) is applied to inhibit neurotransmitter release, and the novel drug Crisugabalin (HSK16149) selectively binds to calcium channels with lower central side effects. Dorsal root ganglion (DRG) radiofrequency ablation, spinal cord stimulation (SCS), and transcutaneous electrical nerve stimulation (TENS) have demonstrated significant efficacy for relieving drug-resistant pain. The recombinant VZV vaccine is more effective than the live-attenuated vaccine in preventing HZ, although long-term safety data remain incomplete. Modulation of the gut microbiota (*e.g*., probiotics and fecal microbiota transplantation) may serve as an adjunctive preventive strategy.

### 3.2 Outlook

Although extensive research has been conducted to investigate the mechanism underlying PHN, no definitive conclusion has been reached. With the development and advancement of technical methods and theories, we anticipate that further studies will be conducted to examine PHN in the future.

#### 3.2.1 Mechanistic Exploration: Association between Viral Latency and Nerve Injury

It is necessary to further elucidate the latency mechanism of herpes zoster virus (HZ) in the nervous system and its dynamic relationship with neuroinflammatory responses and neuronal damage. The causal association between the regulatory pathways of the neuroimmune microenvironment (such as cytokine storm and microglial activation) and neuronal degeneration during viral reactivation should be clarified to provide a theoretical basis for targeted therapy.

#### 3.2.2 Drug Development: Novel Analgesic Strategies with High Efficacy and Low Toxicity

Existing therapeutic drugs exhibit interpatient tolerance differences (*e.g*., central inhibitory effects of anticonvulsants), necessitating the development of new drugs with both high analgesic efficiency and low adverse reactions. Molecular docking and virtual screening technologies (such as natural compound screening targeting the PINK1 pathway in previous studies) can be integrated with emerging targets, such as mitophagy and ion channel regulation, to optimise drug design and enhance clinical efficacy and patient quality of life.

#### 3.2.3 Neuromodulation Techniques: Precision and Efficacy Optimization

Techniques, such as nerve stimulation and spinal cord electrical stimulation, have demonstrated therapeutic potential but require parameter optimisation (*e.g*., stimulation frequency and site selection) through multicentre clinical trials. For example, functional MRI (fMRI)-guided precise localisation of DRG stimulation or closed-loop stimulation systems for dynamic electrical signal regulation can enhance analgesia and reduce the risk of complications.

#### 3.2.4 Immunotherapeutic Interventions: Full-chain Strategies from Prevention to Treatment

Immunotherapy can bidirectionally regulate the PHN course, which includes the following:

- **Preventive Intervention**: It involves optimising the antigen design of recombinant herpes zoster vaccines to enhance immunogenicity and reduce viral reactivation risk.
- **Therapeutic Intervention**: It involves developing monoclonal antibodies targeting proinflammatory cytokines (*e.g*., TNF-α, IL-6) or inhibiting neuroinflammatory cascades by modulating Treg cell function.

#### 3.2.5 Clinical Research: Evidence-driven Improvement of Diagnosis and Treatment Systems

Large-sample, multicentre clinical research should be strengthened to integrate multidimensional data (*e.g*., pain scores such as VAS/NRS, neuroelectrophysiological tests, and imaging indices such as diffusion tensor imaging) to establish a precise diagnosis and treatment model for PHN. Long-term follow-up studies should be conducted to evaluate the long-term safety and efficacy of novel therapies and provide high-level evidence for updating clinical guidelines. As a complex disease, the aetiology and treatment for PHN both remain unclear. By thoroughly reviewing existing research, we can more accurately grasp the focus and difficulties of the current research and provide important guidance and insights for future research. Future research investigating PHN should concentrate on enhancing its efficacy, minimising side effects, improving patient quality of life, and exploring novel treatment strategies and techniques to better comprehend and address the intricate clinical challenges posed by this disease.

## Figures and Tables

**Fig. (4):**
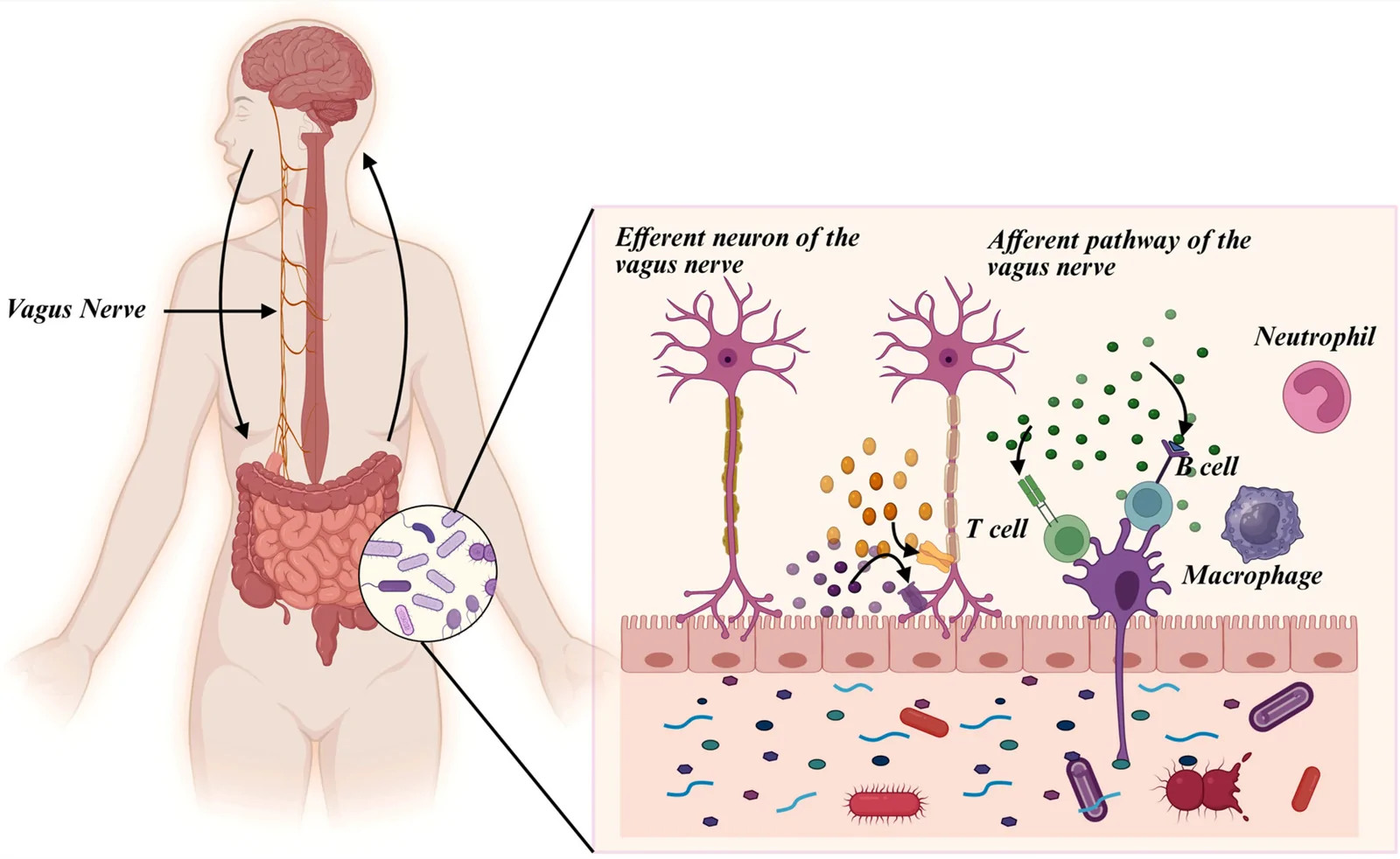
Communication between gut microbiota and the brain is mainly carried out through the gut-brain axis.

**Table 1:** Comparison of drug classification and mechanism.

**Drug Category**	**Representative Drug**	**Core Mechanism of Action**	**Target Site**
Antiviral Drugs	Acyclovir, Valacyclovir	Inhibit VZV DNA replication and reduce nerve damage	VZV polymerase
Calcium Channel Modulators	Pregabalin, Gabapentin	Bind to the α2δ subunit and inhibit Ca^2+^ influx and neurotransmitter release	Voltage-gated calcium channels
Sodium Channel Blockers	Lidocaine Patch	Block Nav1.7/1.8 and inhibit ectopic firing	Peripheral nerve sodium channels
Neurotoxins	Botulinum Toxin A	Inhibit neurotransmitter release, reduce inflammation, and central sensitization	Presynaptic SNARE proteins
Novel Small Molecules	Crisugabalin (HSK16149)	Highly selective binding to α2δ-1 and reduce central side effects	Calcium channel α2δ subunit
Natural Products	Capsaicin, Vitexin	Activate TRPV1 or regulate mitochondrial autophagy	TRPV1 receptor, PINK1 pathway
Immunomodulation	Recombinant VZV Vaccine	Enhances T-cell immunity and prevents virus reactivation	Immune system
Adjuvant Therapy	Magnesium Sulfate, TCAs	Antagonizes NMDA receptors or regulates monoaminergic neurotransmitters	Central nerve receptors/ neurotransmitter systems

## LIST OF ABBREVIATIONS

BMP4: Bone Morphogenetic Protein 4
BoNTs: Botulinum Neurotoxins
BTX: Botulinum Toxin A
CDC: Centers for Disease Control and Prevention
CNS: Central Nervous System
CSF: Cerebrospinal Fluid
CT: Computed Tomography
DMA: Dynamic Mechanical Allodynia
DRG: Dorsal Root Ganglia
EA: Electroacupuncture
FDA: Food and Drug Administration
FMT: Faecal Microbiota Transplantation
GABA: Gamma-aminobutyric Acid
GFAP: Glial Fibrillary Acidic Protein
GM: Gut Microbiota
HSV-1: Herpes Simplex Virus 1
HZ: Herpes Zoster
HZO: Herpes Zoster Ophthalmopathy
IFN-I: Interferon-I
miRNA: MicroRNA
NMDA: N-methyl-D-aspartate
NP: Neuropathic Pain
PHI: Post-herpetic Pruritus
PHN: Post-herpetic Neuralgia
PINK1: PTEN Induced Putative Kinase 1
PRF: Pulsed Radiofrequency
PRMT6: Protein Arginine Methyltransferase 6
ROS: Reactive Oxygen Species
RTX: Resiniferatoxin
lncRNAs: Long Non-coding RNAs
SNI: Spared Nerve Injury
STING: Stimulator of Interferon Gene
TCAs: Tricyclic Antidepressants
TENS: Transcutaneous Electrical Nerve Stimulation
TRPV1: Transient Receptor Potential Vanilloid 1
VAS: Visual Analogue Scale
VSV: Vesicular Stomatitis Virus
VZV: Varicella-zoster Virus
